# The Studies of Prognostic Factors and the Genetic Polymorphism of Methylenetetrahydrofolate Reductase C667T in Thymic Epithelial Tumors

**DOI:** 10.3389/fonc.2022.847957

**Published:** 2022-06-06

**Authors:** Miaolong Yan, Jiayuan Wu, Min Xue, Juanfen Mo, Li Zheng, Jun Zhang, Zhenzhen Gao, Yi Bao

**Affiliations:** ^1^ The Fourth School of Clinical Medicine, Zhejiang Chinese Medical University, Hangzhou, China; ^2^ The Key Laboratory, The Second Affiliated Hospital of Jiaxing University, Jiaxing, China; ^3^ Graduate School, Bengbu Medical College, Bengbu, China; ^4^ The Department of Thoracic Surgery, The Second Affiliated Hospital of Jiaxing University, Jiaxing, China; ^5^ The Department of Oncology, The Second Affiliated Hospital of Jiaxing University, Jiaxing, China

**Keywords:** thymic epithelial tumors, Masaoka–Koga Stage, 8th UICC/AJCC TNM staging, *MTHFR* polymorphism, methylation

## Abstract

**Objective:**

To describe the clinical features of a cohort of patients with thymic epithelial tumors (TETs) and to analyze their prognostic factors. In particular, we investigated the correlation between the genetic polymorphism of methylenetetrahydrofolate reductase (*MTHFR*) C667T and the incidence of TETs.

**Methods:**

Pathological records were reviewed from the database of the Second Affiliated Hospital of Jiaxing University, from January 2010 to December 2020, and 84 patients with TETs were recruited for this study. Univariate and multivariate analyses were performed to determine the prognostic factors. The genetic polymorphism of *MTHFR* C667T was examined in the patients with TETs and in a group of healthy individuals. The correlation between *MTHFR* transcriptional levels and methylation was analyzed using The Cancer Genome Atlas (TCGA) thymoma dataset from the cBioPortal platform.

**Results:**

Kaplan–Meier univariate survival analysis showed that sex, age, the maximum tumor diameter, surgery, chemotherapy, radiotherapy, WHO histological classification, Masaoka–Koga stage, and 8th UICC/AJCC TNM staging, were statistically significantly correlated with the prognosis of patients with TETs. The Masaoka–Koga stage and 8th UICC/AJCC TNM staging were strongly correlated with each other in this study (r=0.925, P<0.001). Cox multivariate survival analysis showed that the maximum tumor diameter, Masaoka–Koga stage, and 8th UICC/AJCC TNM staging were independent prognostic factors affecting the overall survival (OS) of patients with TETs (*P<*0.05). The *MTHFR* C667T genotype (*χ* ^2 =^ 7.987, *P*=0.018) and allele distribution (*χ* ^2 =^ 5.750, *P=*0.016) were significantly different between the patients and healthy controls. CT heterozygous and TT homozygous genotypes at this *MTHFR* polymorphism significantly increased the risk of TETs (odds ratio [OR] *=*4.721, *P=*0.008). Kaplan–Meier univariate survival analysis showed that there was no correlation between different genotypes and the prognosis of TETs (CC versus CT + TT, *χ^2 =^* 0.003, *P*=0.959). Finally, a negative correlation between the transcriptional and methylation levels of *MTHFR* was observed in the TCGA thymoma dataset (*r=*-0.24, *P=*0.010).

**Conclusions:**

The Masaoka–Koga stage, 8th UICC/AJCC TNM staging, and maximum tumor diameter were independent prognostic factors for TETs. Reduced methylation levels of *MTHFR* and particular polymorphic variants may contribute to the susceptibility to developing TETs.

## Introduction

Thymic epithelial tumors (TETs), including thymoma and thymic carcinoma (TC), are derived from thymic epithelial cells and lymphocytes of the thymus. The thymus is the site of maturation for T cells and plays an integral role involving in adaptive immunity. Thymoma is associated with autoimmune-related syndromes including myasthenia gravis (MG), pure erythrocyte hypoplasia, and hypogammaglobulinemia (1, 2). The 5-year survival rates for thymomas and TCs have been reported to be approximately 78% and 40%, respectively (3).

According to the 2015 World Health Organization (WHO) classifications, TETs are classified into six categories: type A, AB, B1, B2, and B3 thymoma and TC, based on the morphology of tumor cells and the number of non-neoplastic lymphocytes (4). Thymomas are divided into two groups: low-risk thymomas (A, AB, and B1) and high-risk thymomas (B2 and B3). The Masaoka–Koga stage is widely adopted for the classification of TETs into stages I, II, III, and IV, based on the degree of tumor cell invasion (5). In addition to the Masaoka–Koga stage, the 8th UICC/AJCC TNM classification is also used in clinical TET staging. This describes not only the degree of tumor invasion but also the tumor spread and lymphatic infiltration (6).

Patients with low-risk thymomas rarely experience recurrence after a complete resection (7), but nevertheless, thymomas are considered as malignant tumors (8). TC is further divided into several subtypes, including squamous cell carcinoma, basaloid carcinoma, mucoepidermoid carcinoma, lymphoepithelioma-like carcinoma, and clear cell carcinoma (9). Most patients with relatively low-risk thymomas are asymptomatic. Conversely, invasive subtypes of thymomas and TCs exhibit aggressive behaviors by invading adjacent structures such as the pleura and pericardium.

Although surgical resection is usually accepted as a primary therapeutic strategy in a curative setting, adjuvant chemotherapy and/or radiotherapy is used in some patients who are at a high risk of recurrence to prolong survival (8, 10).

Currently, the etiology of TETs remains largely unknown. A point mutation in the gene encoding *MTHFR*, which is located on chromosome 1p36.3, is associated with several malignancies, including colon cancer, gastric cancer, lung cancer, esophageal squamous cell carcinoma, breast cancer, and gynecological cancer (11–18). *MTHFR* is a critical enzyme involved in the folate metabolism pathway and DNA methylation and synthesis (19). The *MTHFR* C677T, a cytosine (C) to a thymine (T) substitution at position 677, which changes an alanine to a valine, leads to impaired folate binding and reduced *MTHFR* activity (20, 21). MTHFR deficiency may result in insufficient DNA methylation and genetic instability and may promote the development of malignancies (22).

To explore the correlation between the *MTHFR* C677T polymorphism and TETs, the distribution of this single-nucleotide polymorphism was examined in TET patients and healthy controls in this study. Additionally, the correlation between *MTHFR* methylation and *MTHFR* transcriptional levels was also investigated using The Cancer Genome Atlas (TCGA) database of TETs. A decreased *MTHFR* expression can lead to dysregulated folic acid metabolism, resulting in abnormal DNA methylation and nucleotide synthesis, which may be involved in the susceptibility to develop TETs.

## Materials and Methods

### Study Subjects

We retrospectively analyzed 84 cases of TETs (56 cases of thymoma and 28 cases of TC) who were diagnosed by pathological examination from January 2010 to December 2020 and were treated in the Second Affiliated Hospital of Jiaxing University. Clinical data were recorded and retrieved from the electronic database of the hospital. The present study was approved by the Ethics Committee of the Second Affiliated Hospital of Jiaxing University (Ethical Code number: LWSC041), and written informed consent was obtained from all patients.

### Detection of *MTHFR* Gene Polymorphism and Bioinformatics Study

Among these 84 patients, blood samples were obtained from only 33 cases, which were used to detect *MTHFR* gene polymorphism in this study. Samples were collected from 21 men and 11 women, ranging from 32 to 74 years old between January 2015 and January 2018. A total of 72 healthy individuals (38 men and 34 women) ranging from 51 to 75 years old were recruited as controls. Genomic DNA was extracted from peripheral venous blood (2 ml) using a commercial kit according to the manufacturer’s instructions (BaiO Technology Co, Ltd., Shanghai, China). The DNA purity (A260/A280 ratio) was determined by using a NanoDrop spectrophotometer (Thermo Fisher Scientific, Waltham, MA, USA). The chips that can detect *MTHFR* gene C667T polymorphism were purchased from Shanghai BaiO Company and hybridized by using a BaiO biochip hybridization instrument (BaiO Technology Co., Ltd., China). Data were recorded by a BaiO biochip reader and were analyzed by using a BaiO analysis software (BaiO Technology Co., Ltd., China).

The *MTHFR* transcriptional expression data (123 patients with TETs) from the TCGA database were obtained from the cbioportal platform (http://www.cbioportal.org). Clinical data showed that among these 123 patients with TETs, 64 (52.0%) cases were men and 59 (48.0%) cases were women, with a median age of 60.5 years. Thymectomy was performed in all patients, 41 of whom underwent postoperative radiotherapy and 6 of whom underwent postoperative chemotherapy. The correlation between the mRNA levels of MTHFR and its methylation was analyzed in this platform.

### Treatments

Surgical resection was performed in 76 patients in this retrospective study. The complete resection rate was 77.6%. Among them, 5 patients with incomplete surgical resection underwent multiple therapies. Among a total of 84 patients, 10 patients with thymoma and 22 patients with TC were treated by platinum-based chemotherapy with an average of 4 cycles. A total of 3 patients with thymoma and 6 patients with TC received postoperative adjuvant radiotherapy. The dose of radiotherapy was approximately 54 Gy (1.8 ~ 2.0 Gy/f).

### Follow-Up

The follow-up time was from the time of pathological diagnosis to the death or last follow-up time of the patients, recorded every month. The contents of follow-up included the progression of the disease, stability of the disease, death of the patient, and so on. Overall survival (OS) is defined as the time of the first diagnosis to death. The cut-off time of the study was 2020-12-31.

### Statistical Analysis

All statistical analyses were performed by using Statistical Package for the Social Sciences (SPSS), version 25.0 (IBM Inc., Chicago, IL, USA). Log-rank analysis was used for the comparisons between the different factors affecting prognosis. Multivariate analysis for OS was performed using a Cox proportional hazards model. All significant variables in the univariate analysis including sex, age, smoking status, maximum tumor diameter, chemotherapy, radiotherapy, WHO histological classification, Masaoka–Koga stage, 8th UICC/AJCC TNM staging, and clinical symptoms were used in the multivariate analysis. The Kaplan–Meier survival rate was calculated and drawn by the SPSS software. The chi-square test was used to compare the *MTHFR* C667T genotype and allele distribution between patients with TETs and healthy controls. Logistic regression analysis was used to analyze the correlation between different genotypes *MTHFR* C667T and TET susceptibility. Spearman correlation analysis was performed to analyze the correlations of the mRNA levels of *MTHFR* expression and its methylation by using the TCGA database. *P*<0.05 was considered to be statistically significant.

## Results

### Patient Characteristics

In total, 84 patients diagnosed with thymomas (n=56) and TCs (n=28) who met predefined criteria were recruited in this study. General characteristics were listed in **Table 1**. The study consisted of 51 (60.7%) men and 33 (39.3%) women, with a median age of 58 years (range 22–88 years). In addition, 31 cases were asymptomatic at the first visit, 21 cases complained of chest pain or chest distress, 11 cases had respiratory symptoms (including cough and pulmonary infection), and 12 cases had MG. A total of 9 cases were accompanied by other clinical symptoms, such as lumbago and trauma. At the end of follow-up time on December 31, 2020, 19 patients with TETs were deceased, including 4 patients with thymomas and 15 patients with TCs.

**Table 1:** The characteristics of all the cases recruited in the study (n = 84).

Sex (male)	51 (60.7%)
Age (years)	58 [22,88]
Follow-up duration (months)	36 [1,126]
Medium tumor diameter (cm)	5 [1.1,14.5]
Smoking status	21 (25.0%)
Surgery	76 (90.5%)
Chemotherapy	32 (38.1%)
Radiotherapy	9 (10.7%)
WHO histological classification	
Low-risk thymomas (A/AB/B1)	34 (40.5%)
High-risk thymomas (B2/B3)	22 (26.2%)
Thymic carcinomas (TCs)	28 (33.3%)
Masaoka–Koga stage	
I	23 (27.4%)
II	24 (28.6%)
III	15 (17.8%)
IV	22 (26.2%)
8th UICC/AJCC TNM staging	
I	43 (51.2%)
II	4 (2.8%)
III	15 (17.8%)
IV	22 (26.2%)
Clinical symptoms	
Asymptomatic	31 (36.9%)
Respiratory symptoms	11 (13.1%)
Chest pain/chest distress	21 (25.0%)
Myasthenia gravis	12 (14.3%)
Other symptoms	9 (10.7%)

Respiratory symptoms: cough, pneumonia.

Other symptoms: lumbago, trauma, gout, dizziness, lymphedema.

### Analysis of Prognostic Factors of TETs

**Figure 1:**
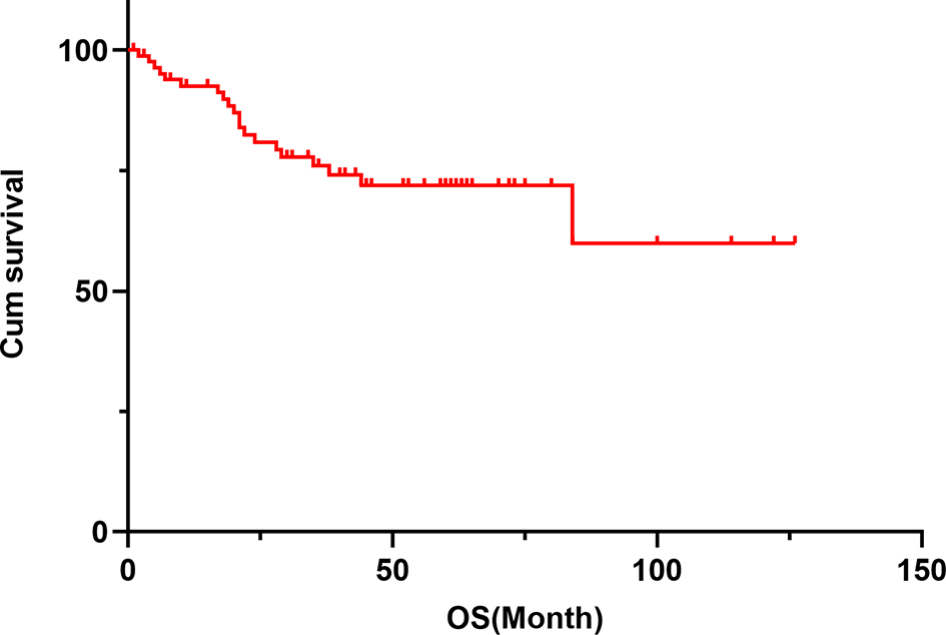
The cumulative survival curve of patients with TETs. The 1-year survival rate was 92.6%, the 3-year survival rate was 76.0%, and the 5-year survival rate was 71.9% in patients with TETs.

**Figure 2:**
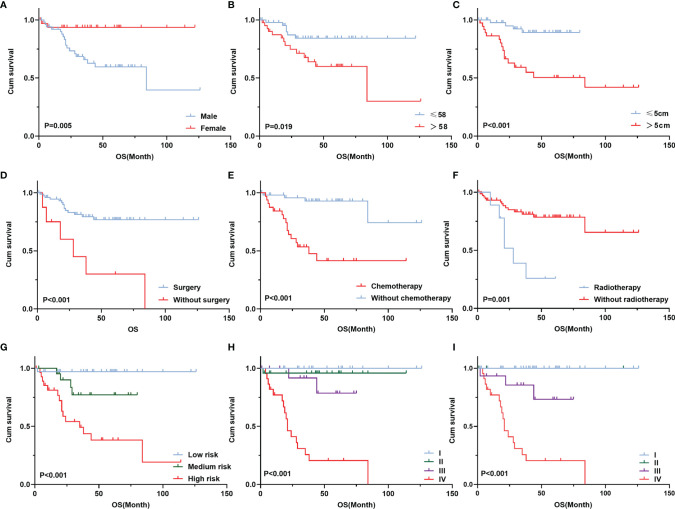
Survival curve of different prognostic factors of 84 patients with TETs. Cumulative survival according to completion of sex **(A)**, age **(B)**, maximum tumor diameter **(C)**, surgery **(D)**, chemotherapy **(E)**, radiotherapy **(F)**, WHO histological classification **(G)**, Masaoka–Koga stage **(H)**, and 8th UICC/AJCC TNM staging **(I)**.

The median follow-up period was 36 months. The 5-year survival rate of patients with TETs was 71.9% (**Figure 1**). Univariate analysis showed that sex (*P* = 0.005), age (*P* = 0.019), the maximum tumor diameter (*P* < 0.001), surgery (*P* < 0.001), chemotherapy (*P* < 0.001), radiotherapy (*P* = 0.001), WHO histological classification (*P* < 0.001), Masaoka–Koga stage (*P* < 0.001), and 8th UICC/AJCC TNM staging TNM stage (*P* < 0.001) were associated with TET prognosis (**Table 2**). Other variables, including clinical symptoms and the smoking status, were not associated to prognosis. Among these factors, male sex, older age (>58 years old), the maximum tumor diameter (>5 cm), no surgery, no chemotherapy, no radiotherapy, a higher grade of WHO histological classification and advanced tumor stage (both the Masaoka–Koga stage and the 8th UICC/AJCC TNM staging) were correlated with poor prognosis. The survival curves of different variables are shown in **Figure 2**: sex (A), age (B), the maximum tumor diameter (C), surgery (D), chemotherapy I, radiotherapy (F), WHO histological classification (G), Masaoka–Koga stage (H), and 8th UICC/AJCC TNM staging (I). The Masaoka–Koga stage and 8th UICC/AJCC TNM staging were strongly correlated with each other in this study (*r*=0.925, *P*<0.001). These univariate analysis data were used to establish a subsequent multivariate Cox proportional hazards regression model on the basis of the Masaoka–Koga stage and UICC/AJCC TNM staging. The results showed that the maximum tumor diameter (*P* = 0.040, hazard ratio [*HR*]: 3.623, *95%CI*: 1.058~12.403 on the basis of Masaoka–Kaga stage; *P* = 0.045, *HR*:3.629, *95%CI*: 1.027~12.822 on the basis of UICC/AJCC TNM staging), Masaoka–Koga stage (*P* = 0.008, HR: 5.513, 95%*CI*: 1.564~19.434), and 8th UICC/AJCC TNM staging (*P* = 0.007, *HR*: 8.476, *95%CI*:1.815~39.596) were identified as independent prognostic factors of patients with TETs (**Tables 3**, **4**).The 5-year survival rates of TET patients with Masaoka–Koga stage I, II, III, and IV were 100%, 95.8%, 78.6%, and 20.5%, respectively (**Figure 2H**). The 5-year survival rates for UICC/AJCC TNM stages I, II, III, and IV were 100%, 100%, 73.3%, and 20.5%, respectively (**Figure 2I**).

**Table 2:** Univariate analyses of overall survival for 84 patients with TETs.

Clinical factors	*χ* ^2^	*P*
Sex		
Female		
Male	7.748	.005
Age (years)		
≤58		
>58	5.460	.019
Maximum tumor diameter (cm)		
≤5		
>5	13.561	.000
Smoking status	1.802	.180
Surgery	13.025	.000
Chemotherapy	19.522	.000
Radiotherapy	10.887	.001
WHO histological classification	24.157	.000
Low-risk thymomas/high-risk thymomas/TCs		
Masaoka–Koga stage	55.816	.000
I/II/III/IV		
8th UICC/AJCC TNM staging	57.096	.000
I/II/III/IV		
Clinical symptoms	5.238	.264
Asymptomatic		
Respiratory symptoms		
Chest pain/chest distress		
Myasthenia gravis		
Other symptoms		

**Table 3:** Multivariate analyses of overall survival for 84 patients with TETs (I).

Clinical factors	HR	95%CI	*P*
Sex			
Female			
Male	1.557	.246-9.836	.638
Age (years)			
≤58			
>58	1.460	.460-4.549	.514
Maximum tumor diameter (cm)			
≤5			
>5	3.623	1.058-12.403	.040
Surgery	1.360	.387-4.784	.632
Chemotherapy	2.160	.550-8.487	.270
Radiotherapy	1.061	.332-3.386	.921
WHO histological classification	1.261	.453-3.508	.657
Low-risk thymomas/high-risk thymomas/TCs			
Masaoka–Koga stage	5.513	1.564-19.434	.008
I/II/III/IV			

**Table 4:** Multivariate analyses of overall survival for 84 patients with TETs (II).

Clinical factors	HR	95%CI	*P*
Sex			
Female			
Male	2.075	.307-14.023	.454
Age (years)			
≤58			
>58	1.333	.416-4.268	.628
Maximum tumor diameter (cm)			
≤5			
>5	3.629	1.027-12.822	.045
Surgery	1.463	.414-5.179	.555
Chemotherapy	1.980	.502-7.818	.330
Radiotherapy	1.032	.332-3.306	.957
WHO histological classification	1.127	.393-3.233	.824
Low-risk thymomas/high-risk thymomas/TCs			
8th UICC/AJCC TNM staging	8.476	1.815-39.596	.007
I/II/III/IV			

### *MTHFR* C667T Genotypes in Patients With TETs

**Figure 3:**
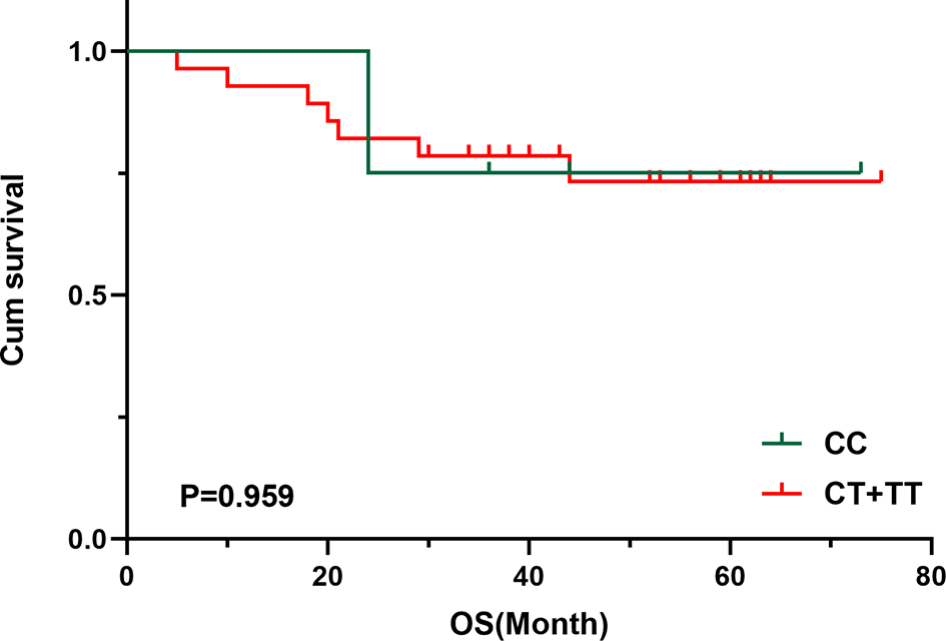
Survival curve of *MTHFR* C667T polymorphism of 33 patients with TETs. Cumulative survival according to *MTHFR* C667T polymorphism.

*MTHFR* polymorphism in healthy individuals and patients with TETs was further investigated in this study. The results of the chi-square test showed that the genotype (*χ* ^2^ = 7.987, *P* = 0.018) and allele distribution (*χ* ^2^ = 5.750, *P* = 0.016) statistically significantly differed between patients and healthy controls (**Table 5**). In logistic regression analysis, heterozygous and homozygous genotypes (CT+TT) showed higher TET susceptibility (odds ratio [*OR*] = 4.721, *P* = 0.008, **Table 6**). The analysis also revealed that, compared to the T allele, the presence of the *MTHFR* C allele at this position was adversely associated with the incidence of TETs (T vs. C: *OR* = 2.067, *95% CI*: 1.136–3.758, *P* = 0.017, **Table 6**). Kaplan–Meier univariate survival analysis was further conducted, and data showed that there was no correlation between different genotypes and the prognosis of TETs (CC vs. CT + TT, *χ* ^2^ = 0.003, *P* = 0.959, **Figure 3**).

**Table 5:** The chi-square test of *MTHFR* C667T genotypes and allele distribution.

Group	Genotype			Allele		*χ* ^2^	*P*
	CC Case (%)	CT Case (%)	TT Case (%)	C Case (%)	T Case (%)		
Controls Patients	29(40.3) 4(12.5)	35(48.6) 22(68.7)	8(11.1) 6(18.8)	93(64.6) 30(46.9)	51(35.4) 34(53.1)	7.987 5.750	.018^a^ .016^b^

a Compared MTHFR C667T genotype distribution in patients with TETs and healthy controls.

b Compared MTHFR C667T allele distribution in patients with TETs and healthy controls.

**Table 6:** Correlation analysis between *MTHFR* C667T genotypes and TET susceptibility.

Variable	OR (95%CI)	*P*
CT/CC	4.557 (1.409-14.735)	.011
TT/CC	2.332 (1.108-4.906)	.026
CT+TT/CC	4.721 (1.497-14.889)	.008
T/C	2.067 (1.136-3.758)	.017

### Correlation of mRNA and Methylation Levels of *MTHFR* in Patients With TETs

**Figure 4:**
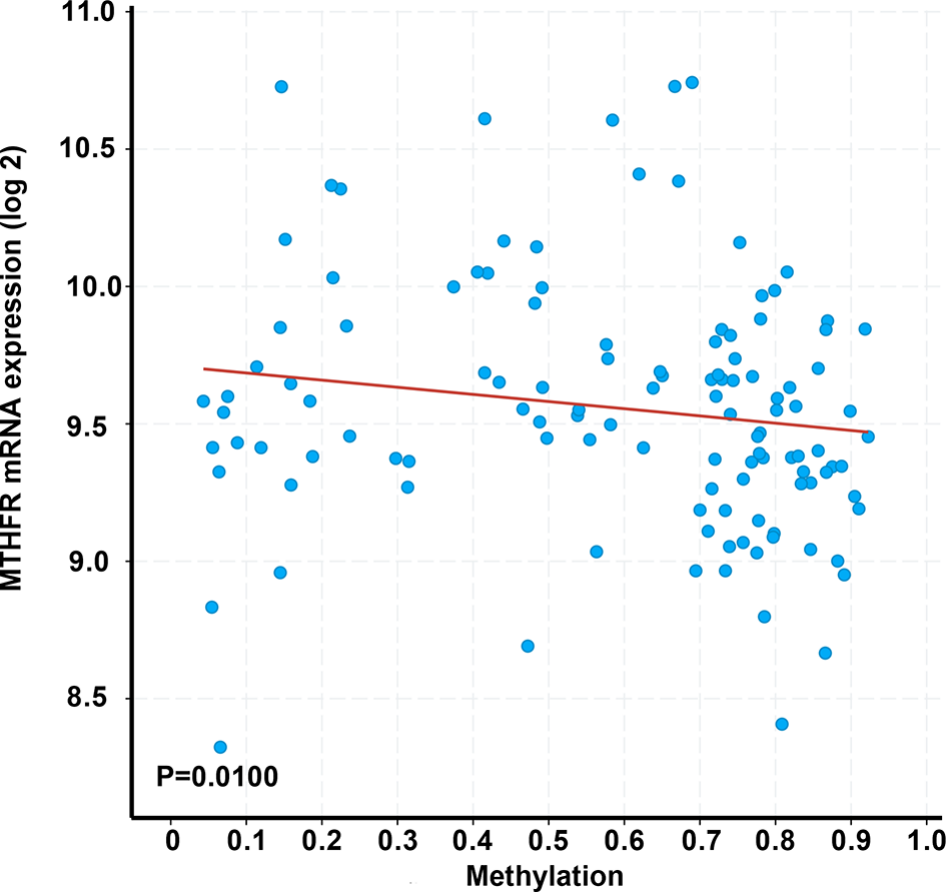
Correlation between *MTHFR* transcriptional levels and its methylation in 123 patients with TETs. Correlations were estimated using Spearman’s bivariate correlation coefficient (*r*= -0.24), with a p-value <0.05 considered as statistically significant.

Using the gene expression profiles of TETs from the TCGA database, a negative correlation was observed between *MTHFR* mRNA levels and their methylation by using Spearman correlation analysis (*r*= -0.24, *P* = 0.010, **Figure 4**).

## Discussion

### Basic Features

In the present study, the median age of the patients at the time of TET diagnosis was 58 years. Using univariate analysis, we observed that the patients with TETs who were older than 58 years or were men had a poor prognosis. As shown in **Figure 1**, the OS of the TET patients gradually decreased with an increase in age. These data were supported by the results of a previous study suggesting that young patients with thymoma have a better performance status and less comorbidities and have a better prognosis than older patients (23). Liu et al. reported similar results in patients with thymoma but not in patients with TC (24). In our study, we analyzed thymoma and TC as a single group, and found that age tended to be a prognostic factor. Sex was not identified as a predictive factor in several studies (25, 26); however, in our study, we observed a worse prognosis in male patients with TETs than in their female counterparts. We consider this to be due to the majority of TC patients in our cohort being men with a reduced survival time.

A tumor diameter greater than 5 cm was observed to be an independent prognostic value of TETs in our study, resulting in a poor prognosis. This conclusion was supported by another study, which showed that the tumor diameter is an independent prognostic factor and may even be an indication for surgical treatment (27). Based on the study by Okumura et al., a tumor diameter ≥5 cm was suggested to be an independent risk factor, which was consistent with our findings (28).

Most patients in our cohort were asymptomatic, which may be related to the improved auxiliary inspection technologies and health awareness in our population. The association between autoimmune diseases (ADs) and thymoma has been well established, particularly MG, which has been accompanied in 20%–25% of thymoma patients based on a previous report (29). In our cohort, approximately 22.9% of thymomas were complicated by MG, but only 7% of TC patients had MG. A further analysis of our data showed that MG did not affect the prognosis of TETs, which was inconsistent with previous studies showing that MG is not an independent or direct prognostic factor of TETs, but it may be helpful in an earlier diagnosis of TETs, leading to a better prognosis (30, 31). However, controversial data showed that patients with MG symptoms had a worse prognosis than patients without MG symptoms (32). The relative small numbers of each cohort due to the low incidence of TETs may contribute to the variance. In addition, the data may present a confounding bias as they were generated from a single center lacking external verification, and therefore, multicenter studies may be needed to draw a comprehensive conclusion.

### WHO Histological Classification

In our study, the cut-off time was 2020-12-31, and therefore, the 2015 WHO histological classification system was used in this study. Our results demonstrated that this classification was associated with the OS of TET patients, but was not an independent prognostic factor by Cox multivariate survival analysis, which was consistent with previous studies (33, 34). An increased WHO histological grade showed more malignant behavior and was associated with a poor prognosis (33). We also reviewed our data using the new 2021 WHO histological classification and observed no change in our results because the new classification system did not affect our data on the pathological diagnosis of TETs (35).

### Masaoka–Koga Stage and 8th UICC/AJCC TNM Staging

The Masaoka–Koga stage was established by Masoka in a retrospective analysis of 93 patients with TETs and was further modified by Koga in 1994 (36, 37). Many studies have shown that the Masaoka–Koga stage is a prognostic factor for predicting the survival time of TETs (38–40). In addition to the Masaoka–Koga stage, the 8th UICC/AJCC TNM staging for TET patients is also used in clinical practice. In our study, both the Masaoka–Koga and the 8th UICC/AJCC TNM staging served as independent prognostic factors for TETs as supported by published literatures (41, 42).

### Treatment

Thymectomy is the first choice of treatment advocated for all patients with TETs and has been shown to be an independent prognostic factor. According to Okereke et al. (43), early-stage thymoma and even advanced TETs with pleural metastasis can benefit from surgical resection (44). In our study, although there was only a correlation in the univariate analysis, the data suggested that most patients who have undergone surgery have a good prognosis. Chemotherapy is not recommended for patients who have undergone complete thymoma resection at an early stage (45). Advanced TETs are prone to invading adjacent tissues and organs, resulting in difficulties associated with incomplete resection. Postoperative adjuvant chemotherapy is recommended in patients with advanced TET to reduce the regional recurrence and distant metastasis (46). Therefore, these patients are likely to benefit from cisplatin-based chemotherapy (47). In our study, the patients with TETs, and apparently those with advanced disease, appeared to benefit from chemotherapy, especially in advanced patients. In addition to chemotherapy, radiotherapy has been proposed to improve the survival of patients with advanced TETs (48). Better outcomes have been observed in patients with TETs receiving postoperative radiotherapy (PORT) than in those who only underwent surgery (48). In our study, patients with TC who were treated with PORT showed remarkable local control with mild toxicities. For patients with advanced TETs, particularly TC patients, it may be valuable to explore novel treatment strategies, such as immunotherapy.

### *MTHFR* C677T Genotypes and Methylation Under TET Patients

Evidence from previous studies implicates the gene encoding the folate-metabolizing enzyme MTHFR in cancer, such as gastric cancer and colorectal cancer (CRC) (49, 50). Our study demonstrates the increased risk of TETs in individuals with heterozygous or homozygous T genotypes (CT+TT) at the C667 polymorphism. Patients with TETs are likely to have CT and TT genotypes, but no correlation was observed between the genetic alteration and prognosis of TETs. According to published data from a meta-analysis of 5,423 CRC patients with CT+TT at the *MTHFR* C667T polymorphism, no prognostic association was observed with either the CC or CT+TT genotypes at the *MTHFR* C667T polymorphism (50). Another study concluded that the *MTHFR* C677T polymorphism is unlikely to be a prognostic factor for esophagogastric cancer (51). These data were consistent with our findings. However, interestingly, data indicated that the *MTHFR* C667T polymorphism affected the prognosis of patients with CRC who were treated with chemotherapeutic drugs including pemetrexed and 5-fluorouracil (52, 53), suggesting that the impaired the folic acid metabolic pathway may affect the sensitivity to chemotherapy drugs and consequent prognostic events.

*MTHFR* C677T polymorphisms decrease the enzyme activity of the *MTHFR* enzyme as compared to the wild type and are associated with DNA hypomethylation (54, 55). This can lead to an increased expression of oncogenes, increased DNA chain breaks, and impaired DNA repair (56). *MTHFR* methylation levels were negatively correlated with the mRNA level of MTHFR using the data derived from the TCGA database. Aberrant DNA methylation may be the etiology and molecular pathogenesis of malignancies including TETs. Chen et al. reported that the degree of hypomethylation was associated with increased disease severity in TETs (1).

## Conclusions

Taken together, in our 84 cases, the maximum tumor diameter (>5 cm), high Masaoka–Koga stage, and 8th UICC/AJCC TNM staging served as independent poor prognostic factors. The decreased methylation of *MTHFR* and increased *MTHFR* C677T genotype (CT+TT) may contribute to the susceptibility to develop TETs.

## Data Availability Statement

The original contributions presented in the study are included in the article/supplementary material. Further inquiries can be directed to the corresponding authors.

## Ethics Statement

The studies involving human participants were reviewed and approved by The ethic committee of the Second Affiliated Hospital of Jiaxing University. The patients/participants provided their written informed consent to participate in this study.

## Author Contributions

MY performed partial clinical data collection, analyses, and manuscript writing. JW performed partial experiments and data analyses. MX, JM, and LZ performed literature reviews and partial data analyses. JZ collected the clinical data of patients with TETs. ZG mentored MY clinical data collection and analyses. YB designed the study and revised the manuscript. All authors read and approved the final manuscript.

## Funding

This work was supported by the Health Bureau of Zhejiang Province (Grant no. 2020KY952), and the Science and Technology Bureau of Jiaxing (Grant no. 2018AY32007 and 2021AY30019).

## Conflict of Interest

The authors declare that the research was conducted in the absence of any commercial or financial relationships that could be construed as a potential conflict of interest.

## Publisher’s Note

All claims expressed in this article are solely those of the authors and do not necessarily represent those of their affiliated organizations, or those of the publisher, the editors and the reviewers. Any product that may be evaluated in this article, or claim that may be made by its manufacturer, is not guaranteed or endorsed by the publisher.
